# Training in Infectious Disease Epidemiology through the Emerging Infections Program Sites

**DOI:** 10.3201/eid2109.150443

**Published:** 2015-09

**Authors:** Duc J. Vugia, James I. Meek, Richard N. Danila, Timothy F. Jones, William Schaffner, Joan Baumbach, Sarah Lathrop, Monica M. Farley, Melissa Tobin-D’Angelo, Lisa Miller, Lee H. Harrison, Nancy M. Bennett, Paul R. Cieslak, Matthew L. Cartter, Arthur L. Reingold

**Affiliations:** California Emerging Infections Program (EIP), California Department of Public Health, Richmond, California, USA (D.J. Vugia) and University of California, Berkeley, California, USA (A.L. Reingold);; Connecticut EIP, Yale School of Public Health, New Haven, Connecticut, USA (J.I. Meek), and Connecticut Department of Public Health, Hartford, Connecticut, USA (M.L. Cartter);; Minnesota EIP, Minnesota Department of Health, St. Paul, Minnesota, USA (R.N. Danila);; Tennessee EIP, Tennessee Department of Health (T.F. Jones) and Vanderbilt University (W. Schaffner), Nashville, Tennessee, USA;; New Mexico EIP, New Mexico Department of Health, Santa Fe, New Mexico, USA (J. Baumbach), and University of New Mexico, Albuquerque, New Mexico, USA (S. Lathrop);; Georgia EIP, Emory University School of Medicine (M.M. Farley), Atlanta Veterans Affairs Medical Center (M.M. Farley), and Georgia Department of Public Health (M. Tobin-D’Angelo), Atlanta, Georgia, USA;; Colorado EIP, Colorado Department of Public Health and Environment, Denver, Colorado, USA (L. Miller);; Maryland EIP, Johns Hopkins University, Baltimore, Maryland, USA (L.H. Harrison);; New York EIP, University of Rochester Medical Center, Rochester, New York, USA (N.M. Bennett);; Oregon EIP, Oregon Health Authority, Portland, Oregon, USA (P.R. Cieslak).

**Keywords:** Emerging Infections Program, training, epidemiology, public health

## Abstract

One objective of the Emerging Infections Program (EIP) of the US Centers for Disease Control and Prevention is to provide training opportunities in infectious disease epidemiology. To determine the extent of training performed since the program's inception in 1995, we reviewed training efforts at the 10 EIP sites. By 2015, all sites hosted trainees (most were graduate public health students and physicians) who worked on a variety of infectious disease surveillance and epidemiologic projects. Trainee projects at all sites were used for graduate student theses or practicums. Numerous projects resulted in conference presentations and publications in peer-reviewed journals. Local public health and health care partners have also benefitted from EIP presentations and training. Consideration should be given to standardizing and documenting EIP training and to sharing useful training initiatives with other state and local health departments and academic institutions.

## Abstract 2

EIP sites contribute substantially to training current and future public health professionals.

The Emerging Infections Program (EIP), funded by the Centers for Disease Control and Prevention (CDC), is a national network for population-based surveillance and epidemiologic studies of emerging infectious diseases in the United States. Since its inception in 1995, the EIP has grown from 4 initial sites to its current network of 10 sites involving state health departments (California, Colorado, Connecticut, Georgia, Maryland, Minnesota, New Mexico, New York, Oregon, and Tennessee) and collaborators in academic institutions, local health departments, health care facilities, and clinical laboratories, as well as in CDC and other federal agencies (*1*). One of the objectives of the EIP is to “Provide training opportunities in infectious disease epidemiology…” (*2*). Training opportunities were to be based on EIP activities, primarily active or enhanced surveillance and applied research on the prevention and control of emerging infectious diseases, most of which fall under the rubrics of invasive bacterial diseases, foodborne diseases, influenza, and health care–associated infections (*1*). Because there has been no dedicated funding or standard guidelines for the EIP training objective, each site has determined what training to provide, to whom, and how.

Most EIP sites are directed by a partnership of co-directors from a state health department and a local/regional school of public health or school of medicine, to maximize the strengths of both institutions. Many senior EIP staff at state health departments hold voluntary faculty appointments at their local schools of public health or medicine. In addition, each EIP site collaborates extensively with its local health departments, health care facilities, clinical laboratories, and other nearby academic institutions. We contacted all 10 EIP sites to ascertain the extent of training performed during the first 2 decades of the program and develop recommendations for further improving these activities as the program moves forward.

## EIP Trainees and Training Opportunities

By the 20th year of the EIP, all 10 sites had hosted a variety of trainees. Not all sites have consistently documented all training activities, but adequate information was available to provide an overall picture of the types of trainees and the spectrum and depth of their activities. Trainees have included undergraduates; graduate students (candidates for master of public health [MPH], doctor of public health, doctor of philosophy, and doctor of medicine degrees); postgraduate fellows; medical residents or infectious disease fellows; CDC Epidemic Intelligence Service officers; and laboratory personnel. Most trainees came from local schools of public health or medicine, but many also came from distant institutions (including international). Connecticut EIP trainees, for example, have come from 18 different colleges and universities, with most from the Yale School of Public Health.

All 10 EIP sites have had trainee projects that were used for graduate student theses or practicums. At the Connecticut EIP, >190 students received training from 1995 through 2014. Of these, 75 used their experiences to fulfill thesis requirements, and 29 published their work in a peer-reviewed journal. Similarly, at the Minnesota EIP, 116 master’s theses and 7 doctoral theses were written on the basis of EIP data, and at least 15 of these were subsequently published in peer-reviewed literature. Examples of EIP surveillance and epidemiologic projects on which trainees have worked illustrate the wide variety of emerging infectious disease issues and datasets available to trainees (Table). Projects have included site-specific data as well as data from several participating EIP sites.

**Table:** Examples of surveillance activities and epidemiologic projects involving trainees, Emerging Infections Program sites, United States, 1995–2014

Surveillance and epidemiologic projects
A. Invasive bacterial diseases
a. Invasive pneumococcal disease
b. Pneumococcal conjugate vaccine effectiveness
c. Pneumococcal carriage
d. Invasive group B streptococcal disease
e. Invasive group A streptococcal disease
f. *Neisseria meningitidis* infections
B. Foodborne diseases
a. *Salmonella* infections
b. *Salmonella* antibiotic resistance
c. *Shigella* infections
d. *Campylobacter* infections
e. Shiga toxin–producing *Escherichia coli* (STEC), O157, and non-O157
f. *Cryptosporidium* infections
C. Health care–associated infections
a. Methicillin-resistant *Staphylococcus aureus* (MRSA) infections
b. *Clostridium difficile* infections
D. Influenza
a. Influenza surveillance
b. Influenza A(H1N1) hospitalizations
c. Guillain-Barré syndrome surveillance
E. Other diseases or conditions
a. Unexplained illness and death surveillance
b. Fungal infection surveillance
c. Tickborne disease surveillance
d. Acute/chronic liver disease surveillance
e. Encephalitis etiology
f. Human papillomavirus vaccine effectiveness

Undergraduate and graduate students have also been employed on a part-time or short term basis at several EIP sites. These students typically worked on implementing EIP surveillance activities and epidemiologic investigations, including data collection, entry, analysis, and reporting. Many EIP trainees have subsequently entered the public health workforce at the local, state, and federal levels (including CDC and the Food and Drug Administration), and some have become permanent employees at the sites where they trained. Others have gone on for additional study or have taken positions in hospitals and academia.

### Symposia/Regional Conferences

Most EIP sites regularly provided scientific presentations, symposia, and updates on emerging infectious diseases to local health care and public health partners. For example, the Minnesota EIP has sponsored 20 annual 1- or 2-day conferences on “Emerging Infections and Clinical Medicine,” with an average of 275 attendees each year. In fall 2014, the Tennessee EIP conducted its 15th Annual Scientific Presentation Day program, hosting ≈300 attendees from across Tennessee, and the California EIP held its 14th annual “Under Surveillance” symposium with 131 attendees from the San Francisco Bay area. In March 2015, the Georgia EIP hosted its 12th Annual EIP Meeting, with ≈230 attendees. Attendees served by these regional conferences have included public health nurses, epidemiologists, laboratorians, hospital infection control practitioners, students, and health care providers.

### Examples of Local Training Activities

#### Connecticut EIP

Connecticut EIP staff from the state health department and Yale EIP co-teach a full semester seminar course, “Investigation of Disease Outbreaks,” for MPH students at the Yale School of Public Health; 257 students took the course during 1999–2014. This popular practical course on applied field epidemiology highlights many of the innovative surveillance and analytic epidemiology methods developed by the EIP network. EIP staff have collaborated with the Connecticut Department of Public Health’s Food Protection Program to provide a variety of training opportunities to local health departments on topics that included foodborne disease surveillance and outbreak detection, investigation, and response. EIP staff have served as speakers at annual statewide environmental health training programs and regional recertification training workshops for local sanitarians. In 2011 and 2013, EIP staff provided training in outbreak response to a multidisciplinary audience comprised of public health nurses, sanitarians, laboratorians, and epidemiologists, using the Council to Improve Foodborne Outbreak Response Toolkit (*3*).

#### Georgia EIP

Since 2011, the Georgia EIP has offered a 1-year fellowship for infectious disease fellows in their third year at the Emory University School of Medicine. Under the supervision of the Georgia EIP co-director at Emory, the fellow is trained in the use of SAS statistical software and other analytic tools and is expected to present study results at a regional or national meeting and submit a manuscript to a peer-reviewed journal.

#### New Mexico EIP

New Mexico EIP staff have helped develop and implement a curriculum for second-year medical students that provides a service-learning opportunity in infection control and prevention in outpatient settings. A pilot study conducted in 2013 (and an expanded offering in 2014) involved 19 medical students with the following results: 1) increased medical student awareness and knowledge of infection control practices and their role in the ambulatory care setting, 2) provided feedback to the practices concerning quality improvement recommendations, and 3) increased awareness among community health settings of best practices in infection control.

#### Tennessee EIP

Tennessee EIP staff have provided annual outbreak training to public health personnel statewide for >14 years. Training exercises have frequently focused on pathogens and diseases being monitored by EIP and have included hands-on training in the evaluation of surveillance systems and in outbreak detection, investigation, and response. Trainees have included nurses, epidemiologists, laboratorians, and environmentalists, with attendance ranging from 100 to 250 each year. Beginning in 2010, Tennessee EIP FoodNet staff have conducted a course for MPH students at Vanderbilt University on public health surveillance, focusing primarily on EIP-related topics.

## Training Contributions of EIP Sites

The EIP has made substantial contributions to the training objective in CDC’s plan to address emerging infectious diseases in the coming century (*2*). A strength common to EIP sites is the level of engagement of the involved health departments and universities in using epidemiology to address practical questions of public health importance. EIP trainees enjoy the mentorship of academicians and governmental public health practitioners and have a foundation on which to hone skills in disease surveillance, data systems, descriptive and analytic epidemiology, and, in many cases, shaping policy.

The EIP provides a unique opportunity for students at all levels to experience real-world, applied public health, in the context of their academic training. Trainees find it invaluable to participate personally and collaboratively in all levels of a public health activity, from hypothesis generation and data collection to data analyses and final drafting of a report. The training provided by EIP sites is on-the-job training, usually with a one-on-one mentoring relationship between trainee and supervisor. Training capacity is frequently limited by the number of principal investigators and supervisors available to serve as mentors. The large amount of time dedicated to working with trainees is a testament to the commitment that EIP sites make to training the next generations of health care and public health professionals.

Thousands of local public health and health care partners have benefitted from annual local EIP symposia and presentations. The symposia have provided valuable continuing education and opportunities for local and state public health and health care professionals to meet and share experiences as they address critical issues in their communities. The symposia also illustrate how data collected locally can be used to create national public health policy.

## Strengthening and Expanding EIP Training

As the EIP continues to carry out its public health mission, reevaluating its training objective and building on past successes will be essential. Efforts to standardize, network, and share training opportunities can strengthen and expand the EIP training objective to benefit future public health professionals through public health service and research on emerging infectious diseases.

EIP training activities should be systematically documented at all sites in a standardized manner, and EIP trainees should be asked to provide a formal evaluation of their training experience. Standardized documentation of these training experiences will allow future evaluation and potential improvement benefitting trainees and supervisors, as well as the partner institutions involved. Such objective assessments can be used to document the utility for dedicated funding to support the training mission of the EIP network.

Several EIP sites have developed additional training initiatives that involve implementing projects specific to their site, to the benefit of local public health and health care students and professionals. Efforts should be made to share these experiences among EIP sites and with non-EIP state health departments, many of whom already partner with local schools of public health or medicine. Expansion of similar trainings in non-EIP sites could be implemented with moderate funding support.

In conclusion, EIP sites have contributed, and will continue to add, to the training of current and future public health and health care professionals, using EIP population-based surveillance activities and projects on emerging infectious diseases. Consideration should be given to standardizing and documenting EIP training activities and to sharing useful training initiatives with other state and local health departments and academic institutions. Such efforts can contribute further to the training of the next generation of the nation’s public health and epidemiology workforce.
